# TMEM140 is associated with the prognosis of glioma by promoting cell viability and invasion

**DOI:** 10.1186/s13045-015-0187-4

**Published:** 2015-07-22

**Authors:** Bin Li, Ming-Zhu Huang, Xiao-Qiang Wang, Bang-Bao Tao, Jun Zhong, Xu-Hui Wang, Wen-Chuan Zhang, Shi-Ting Li

**Affiliations:** Department of Neurosurgery, Xinhua Hospital, Shanghai Jiaotong University School of Medicine, Shanghai, 200092 China; Department of Oncology, Fudan University Shanghai Cancer Center, Shanghai, 200032 China

**Keywords:** TMEM140, Glioma, Cell viability, Invasion, Prognosis

## Abstract

**Background:**

Gliomas are the most common types of primary brain tumors in the adult central nervous system. TMEM140 is identified as an amplified gene in the human gastric cancer genome. However, the function of TMEM140 in gliomas has not been thoroughly elucidated. The aim of the current study was to determine the clinical significance of TMEM140 expression in patients with gliomas and its effect on tumor cell malignant phenotypes.

**Methods:**

Immunohistochemical analysis and real-time reverse transcription PCR were performed to detect the expression levels of TMEM140 in 70 glioma brain tissue samples. Next, the correlation between the TMEM140 expression levels and the clinical characteristics and outcomes of glioma patients was statistically analyzed. TMEM140 expression was inhibited in two glioma cell lines (i.e., U87 and U373) using a knockdown method with small interfering RNA. Cell Counting Kit-8 and Transwell assays were used to investigate TMEM140 function during cell proliferation, invasion, and migration, respectively. Using flow cytometry and Western blot analysis, we subsequently determined the cell cycle and apoptosis profile of the TMEM140-silenced cells.

**Results:**

TMEM140 protein expression was significantly higher in gliomas than in normal brain tissues (*p* < 0.0001). TMEM140 overexpression was strongly correlated with tumor size, histologic grade, and overall survival time (*P* < 0.05). TMEM140 decreased cell viability in vitro and dramatically decreased tumor volume in vivo. This phenomenon might be caused by G1 phase cell cycle arrest and cell apoptosis. TMEM140 silencing could suppress the viability, migration, and invasion of glioma cells.

**Conclusions:**

Our results suggest that TMEM140 expression is a prognostic factor that might play an important role in the viability, migration, and invasion of glioma cells. This study highlights the importance of TMEM140 as a novel prognostic marker and as an attractive therapeutic target for gliomas.

## Background

Gliomas, which arise from glia cells, are the most common type of primary tumors in the central nervous system. Malignant glioma is classified as grade II–IV according to the 2007 World Health Organization (WHO) classification system [1]. Malignant glioma is a leading cause of death in patients, accounting for 45–55 % of primary intracranial tumors. The annual incidence of malignant glioma is greater than 5 per 100,000 population [2]. Grade IV glioblastoma (GBM) is the most common and biologically aggressive malignant glioma. It is characterized by the hallmark features of unrestrained cellular proliferation, strong resistance to apoptosis, diffuse infiltration, vigorous angiogenesis and widespread genomic instability. With advances in aggressive surgery, radiation, and chemotherapy, the clinic survival rate for glioma has been greatly improved [3–5]. However, the median survival rate of patients with GBM remains less than 1 year [6, 7]. Understanding the molecular mechanisms involved in the formation and development of glioma is important for preventing and treating glioma and improving patient survival. Numerous molecular abnormalities, such as gene sequence alterations, DNA copy-number changes, chromosomal rearrangements, DNA methylation, and abnormal regulation of signaling pathways, have been associated with the tumorigenesis and development of glioma [8]. Inactivation of the Rb and p53 pathway [9–11], activation of receptor tyrosine kinases (RTKs) [8], and EGFRvIII activity [12], which are involved in tumor cell adhesion, migration, invasion, proliferation, and apoptosis, are observed in glioma [13–16]. However, the exact mechanism associated with this relationship remains unclear [15].

Transmembrane protein 140 (TMEM140, also known as FLJ11000) is a 185 amino acid protein encoded by a gene that maps to chromosome 7q33. Few studies have investigated the functions of this protein. Guan et al. reported an inhibitory effect of TMEM140 on herpes simplex virus 1 (HSV-1) proliferation [17]. Although TMEM140 has been identified as an amplified gene in the human gastric cancer genome [18], to the best of our knowledge, there have been no reports describing the functions of TMEM140 in tumorigenesis and development of tumors. The present study demonstrated the overexpression of TMEM140 in glioma tissues and analyzed the prognostic significance of TMEM140 expression in a large number of patients with gliomas. Next, we then investigated the effects of TMEM140 knockdown on cell viability and invasion in vitro and in vivo. Furthermore, we explored the underlying mechanisms. Our data suggest that TMEM140 can be a novel prognostic factor and potential treatment target for gliomas.

## Results

### Overexpression of TMEM140 and its prognostic significance in glioma patients

**Fig. 1:**
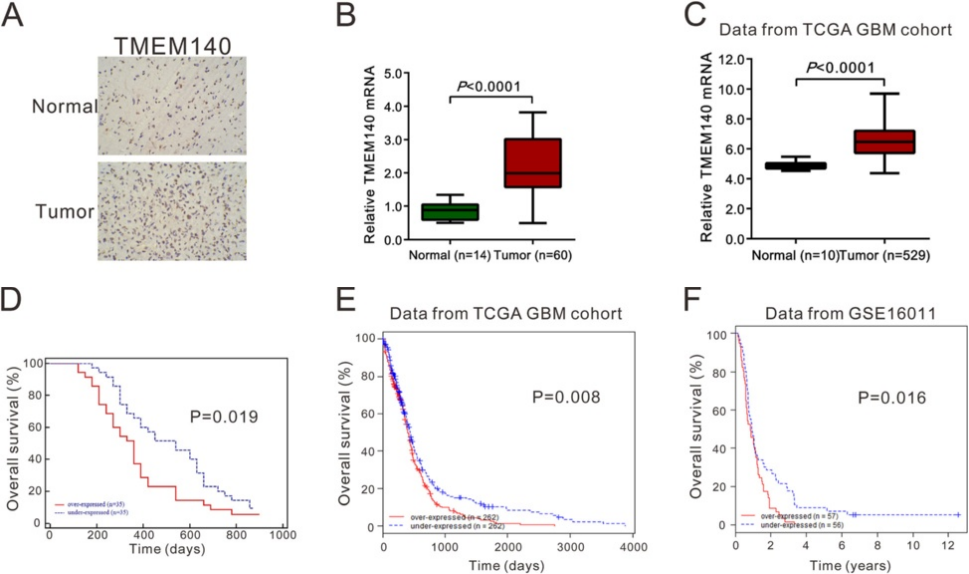
TMEM140 was overexpressed in glioma and was negatively correlated with patient survival. **a** IHC staining of human glioma tissues using TMEM140-specific antibody (brown staining), as described in “Material and methods” section. **b** Quantification of TMEM140 mRNA expression in glioma and normal brain tissues. **c** TMEM140 expression was significantly increased in glioma tissues (*n* = 529) compared with normal tissues from the patients (*n* = 10) from the TCGA GBM dataset (*P* < 0.0001). Survival analysis of patients using our own data (**d**, *P* < 0.05), as well as the TCGA GBM dataset (**e**, *P* < 0.01) and GSE16011 dataset (**f**, *P* < 0.05)

To investigate the protein expression profile of TMEM140 in gliomas, immunohistochemistry (IHC) was used in 70 formalin-fixed, paraffin-embedded tissue sections and 14 non-neoplastic brain tissues. As shown in Fig. 1a, TMEM140 can be observed in 67.1 % (47/70) of the glioma specimens. Note that the control brain tissues had lower expression levels of TMEM140 protein compared with the glioma tissues.

We quantified the expression level of TMEM140 in glioma tissues compared with the control brain tissues (60 gliomas and 14 controls) using real-time reverse transcription PCR (RT-PCR) analysis. As shown in Fig. 1b, the level of TMEM140 in the tumor tissues was significantly higher than that in the controlled brain tissues (2.21 ± 0.11 vs. 0.87 ± 0.07, respectively, *P* < 0.0001). Next, we reanalyzed the high throughput RNA-sequencing data from the GBM cohort of the Cancer Genome Atlas (TCGA), and we found that TMEM140 expression was significantly increased in glioma tissue compared with normal brain tissue (Fig. 1c, *P* < 0.001).

The relationship between the clinicopathologic features and TMEM140 protein expression levels in 70 glioma patients is summarized in Table 1. A chi-square test showed that the increased expression level of TMEM140 was significantly correlated with tumor size (*P* = 0.0211) and pathological grade (*P* = 0.0108). As shown in Table 1, there was no correlation between the expression level of TMEM140 protein and patient age or gender.

**Table 1:** Correlation of TMEM140 expression in human glioma patients with different clinicopathological features (*n* = 70)

Parameters	Characteristic	TMEM140		*P* value
		High (*n* = 47)	Low (*n* = 23)	
Age (years)	≥55	27	13	0.6164
	<55	18	12	
Gender	Male	16	10	0.5990
	Female	31	13	
Tumor size	≥4.5 cm	20	17	0.0211*
	<4.5 cm	27	6	
WHO grade	I/II	15	15	0.0108*
	III/IV	32	8	

*WHO* World Health Organization

*P<0.05

Therefore, we analyzed the relationship between TMEM140 protein overexpression and patient prognosis. A Kaplan-Meier survival curve was used to analyze the prognostic significance of TMEM140 expression. TMEM140 expression was divided into high-expression and low-expression groups. It was observed that patients with gliomas expressing high levels of TMEM140 showed statistically poorer prognoses compared with patients with gliomas expressing low levels of TMEM140- (*P* = 0.019; Fig. 1d). Furthermore, analyses of the TCGA GBM (https://tcga-data.nci.nih.gov/tcga/tcgaCancerDetails.jsp?diseaseType=GBM&diseaseName=Glioblastoma%20multiforme) and GSE16011 datasets [18] (http://www.ebi.ac.uk/arrayexpress/experiments/E-GEOD-16011/?query=GSE16011) also showed that patients with high TMEM140 protein expression had a significantly shorter overall survival rate time (TCGA, *P* = 0.008; GSE16011, *P* = 0.016; Fig. 1e, f).

### TMEM140 silencing suppressed the growth of glioma cells in vitro and in vivo

**Fig. 2:**
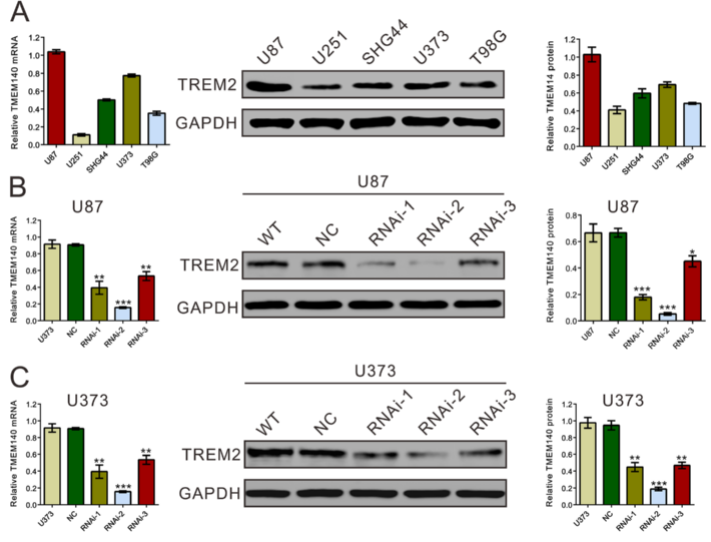
Suppressing of TMEM140 expression by RNAi. **a** TMEM140 expression level in five glioma cell lines was analyzed by RT-PCR (*left*) and immunoblot (*middle* and *right*). **b**, **c** The effect of TMEM140 knockdown through siRNA silencing. The cells were transfected with normal control or TMEM140-RNAi for 48 h and then subjected to RT-PCR (*left*) and immunoblot analysis (*middle* and *right*) of the TMEM140 expression level. The representative images for immunoblot are shown in the *middle panel*, and data from three independent experiments were expressed as the mean ± S.D. (*right panel*). Wild type: wild-type cells; normal control: scrambled siRNA transfected cells; RNAi-1, RNAi-2, and RNAi-3: TMEM140-RNAi-1, -2, and -3 transfected cells (*P < 0.05, **P < 0.01, ***P < 0.001 compared with normal control)

To address the efficacy of TMEM140 on glioma cells, we knocked down TMEM140 in the glioma cell lines. We evaluated the expression levels of TMEM140 in five glioma cell lines (i.e., U87, U251, SHG44, U373, and T98G) through RT-PCR and Western blot analysis (Fig. 2a). Two cell lines (U87 and U373) that showed higher TMEM140 mRNA and protein expression levels were selected for RNA interference experiments. As shown in Fig. 2b, c, all three TMEM140 small interfering RNA (siRNA) sequences showed efficient silencing of TMEM140 expression based on Western blot and RT-PCR analyses. TMEM140-RNAi-2 was the most effective protein, and it was used for the following assays.

**Fig. 3:**
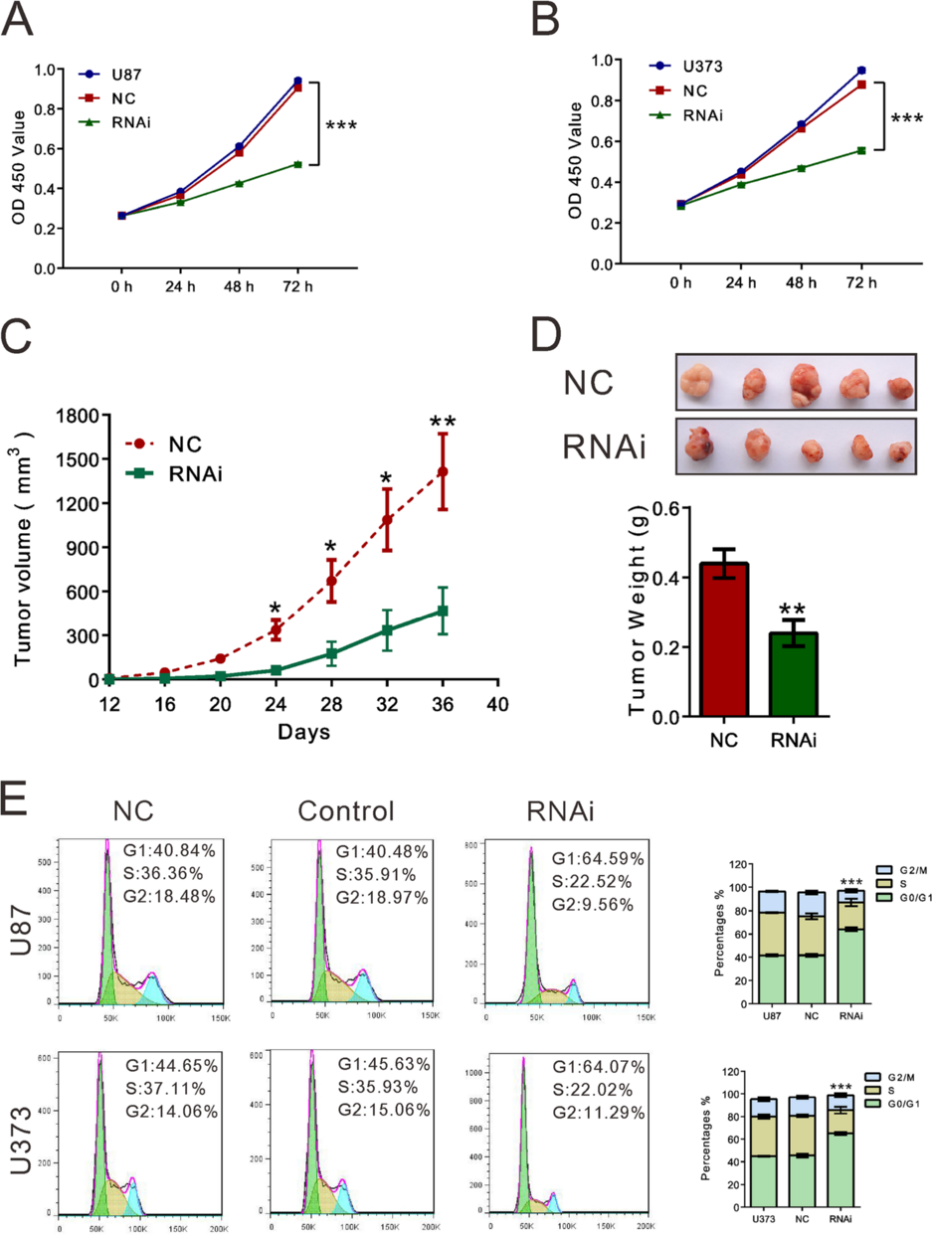
The growth-suppressive effect of TMEM140 silencing on GBM cells in vitro and in vivo*.* **a**, **b** The cells were transfected with TMEM140-RNAi-2 or normal control and then subjected to cell proliferation assay by CCK-8. TMEM140 knockdown via siRNA silencing inhibited the proliferation of U87 (**a**) and U373 (**b**) cells. **c**, **d** TMEM140 knockdown inhibited the growth of U87 cells in vivo. The representative images for xenograft tumor on the nude mouse are shown in Fig. 1d (*top panel*), and the tumor growth curve or tumor weight is shown in Fig. 1c, d (*n* = 5). **e** U87 and U373 cells were transfected with the indicated siRNA. Cells were collected after 48 h, and cell cycle profile was analyzed using flow cytometry. The representative images are shown in the *left panel*, and data from three independent experiments are expressed as the mean ± S.D. (*right panel*) (*P < 0.05, ** *P* < 0.01, ***P < 0.001 compared with normal controls)

Cell growth analysis was performed for two glioma cell lines (U87 and U373) transfected with TMEM140-RNAi-2, which were then subjected to cell growth analysis. In a cell viability assay, both glioma cell lines showed significant reductions in cell viability through TMEM140 silencing compared with normal control cells (Fig. 3a, b). Seventy-two hours after siRNA transfection, cell viability was reduced 42.3 and 36.8 % in the U87 and U373 cells, respectively.

We also found that the in vivo tumor growth and weight in the TMEM140-RNAi group were strongly inhibited compared with the normal control group (Fig. 3c, d). At the end point, the tumor volumes were 498.4 ± 62.0 and 1145.1 ± 145.4 mm^3^ in the TMEM140-RNAi and normal control groups, respectively (*P* < 0.01). Furthermore, the tumor weights were 0.44 ± 0.04 and 0.24 ± 0.04 g in the TMEM140-RNAi and normal control groups, respectively (*P* < 0.01). Therefore, these findings showed that TMEM140 was required for the growth of glioma cells both in vitro and in vivo.

To address the mechanism underlying the growth suppression of glioma cells by TMEM140 knockdown, we then determined the cell cycle profile (Fig. 3e) of the TMEM140-silenced cells through flow cytometry. The results showed that TMEM140 silencing increased the population of the U87 and U373 cells in the G0/G1 phase by 53.5 and 43.3 %, respectively (*P* < 0.001).

### TMEM140 silencing caused apoptosis in the glioma cells

**Fig. 4:**
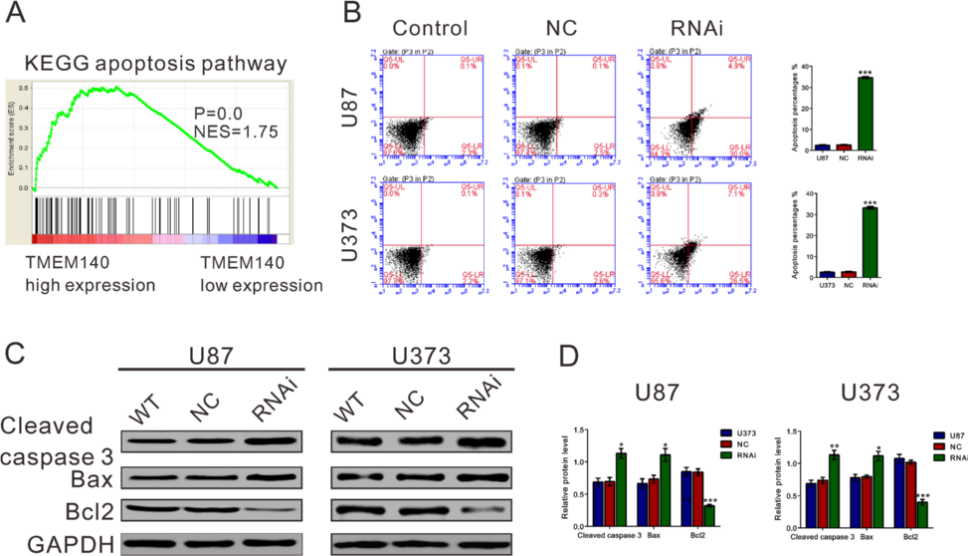
Depletion of TMEM140-induced apoptosis of glioma cells. **a** GSEA was performed using the TCGA GBM dataset. The cell apoptosis pathway was positive associated with TMEM140 higher expression. **b** U87 and U373 cells were transfected with indicated siRNA. Cells were harvest after 48 h, double labeled with Annexin V-FITC and PI, and analyzed using flow cytometry. **c, d** The expression levels of three apoptotic factors (cleaved caspase, Bax and Bcl2) were evaluated by western blot analysis. The representative images are shown in the *left panel*, and data from three independent experiments are expressed as the mean ± S.D. (*right panel*) (*P < 0.05, ** *P* < 0.01, ***P < 0.001 compared with the normal controls)

**Fig. 5:**
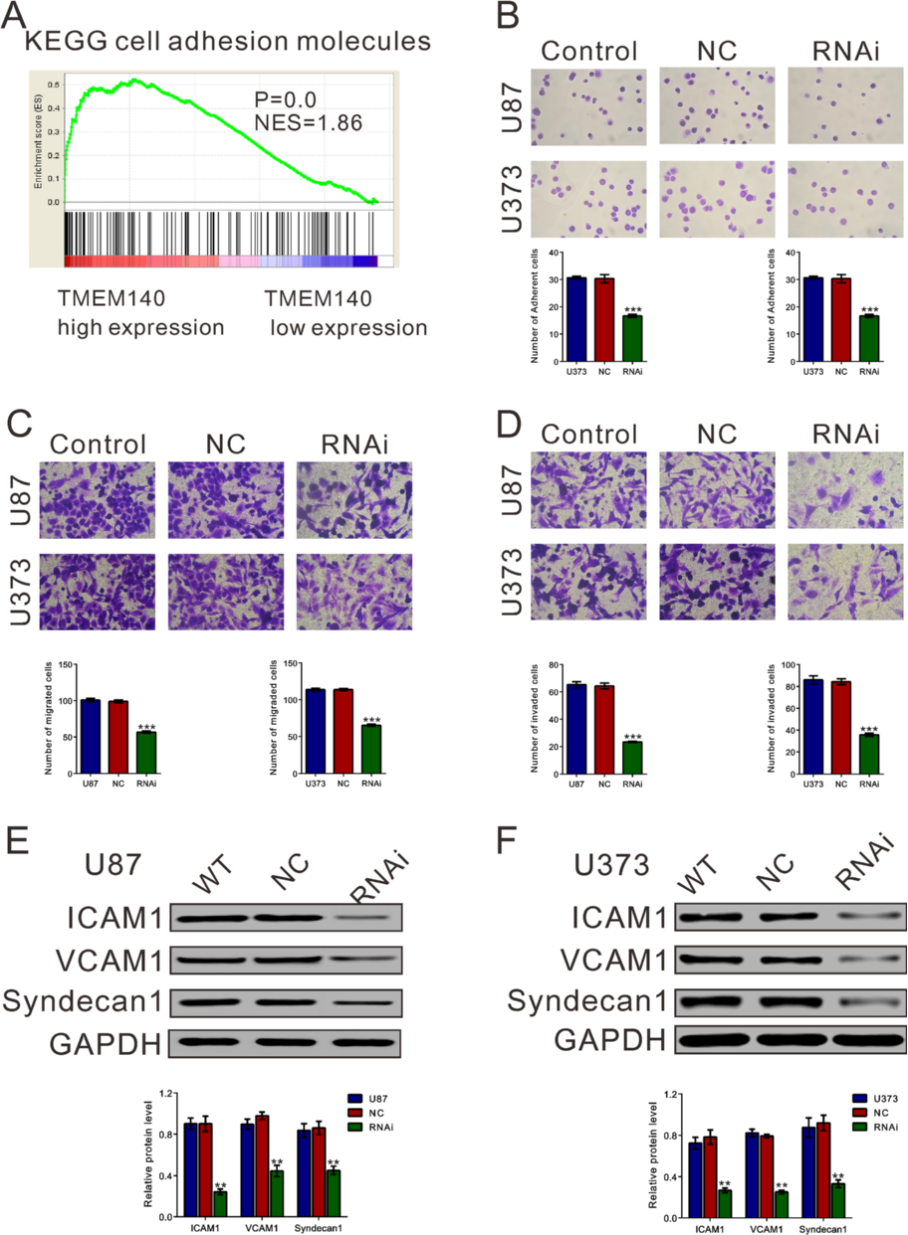
TMEM140 silencing suppressed adhesion, migration, and invasion in the glioma cells. **a** Based on the TCGA GBM dataset, GSEA showed that KEGG cell adhesion molecules were positively associated with higher TMEM140 expression levels. The cells were transfected with normal control or TMEM140-RNAi for 48 h and subjected to cell adhesion, migration, and invasion assay. **b** TMEM140 knockdown inhibited glioma cell adhesion. **c** TMEM140 knockdown inhibited the migration abilities of glioma cells. **d** TMEM140 knockdown suppressed the invasive abilities of the glioma cells. The representative images are shown in the *upper panel*, and data from three independent experiments are expressed as the mean ± S.D. **e, f** Expression levels of cell adhesion molecules, ICAM1, VCAM1, and Syndecan1 were evaluated by Western blot analysis (*lower panel*). (*P < 0.05, **P < 0.01, ***P < 0.001 compared with the normal controls)

To probe the TMEM140-associated pathways in an unbiased manner, we performed Gene Set Enrichment Analysis (GSEA) based on the TCGA GBM dataset. We found that the KEGG apoptosis pathway (Fig. 4a) and cell adhesion molecules (Fig. 5a) were identified with the strongest association with higher TMEM40 expression levels.

The effect of TMEM140 on apoptosis in glioma cells (Fig. 4b) was investigated. TMEM140 knockdown in the glioma cells induced cell apoptosis at approximately 12-fold the rate in the corresponding normal control cells. Moreover, three apoptotic regulation factors [19] (i.e., Bcl2, Bax, and cleaved caspase3) were evaluated using Western blot analysis. TMEM140 deficiency caused the up-regulation of the proteins promoting apoptosis (Bax and cleaved caspase3) and the down-regulation of the protein inhibiting apoptosis (Bcl2) (Fig. 4c, d).

### TMEM140 silencing diminished the adhesion, migration and invasion of glioma cells

Next, we next investigated the effects of TMEM140 on cell adhesion, migration, and invasion. TMEM140 knockdown decreased the adherent U87 and U373 cells by 61.5 and 43.3 %, respectively (Fig. 4b). TMEM140 knockdown strongly inhibited cell motility, with fewer than 60 % of the U87 and U373 cells migrating (Fig. 4c). In contrast to the normal control cells, the invasive ability of the TMEM140 knockdown cells was greatly reduced (Fig. 4d). The number of invaded U87 and U373 cells with TMEM140 silenced was 35.9 % compared with 42.9 % for the normal control cells.

In contrast with the normal control cells, knockdown TMEM140 caused significantly down-regulated cell adhesion molecules (ICAM1, VCAM1, and Syndecan1) in the U87 and U373 cells. This finding is also consistent with functional characterization in vitro.

## Discussion

TMEM140 gene is located on chromosome 7, and alterations of chromosome 7 are closely related to various cancers [20–25], such as breast cancer, prostate cancer, and gliomas. It remains unclear whether TMEM140 has important biological functions in tumors. In the present study, we found that TMEM140 was frequently overexpressed in 67.1 % (47/70) of the glioma tissues. We hypothesized that the overexpression of TMEM140 may promote tumor cell growth in gliomas. To test this hypothesis, we examined the expression status of TMEM140 and the clinicopathologic features, as well as the biological significance of its expression in glioma cell lines. Consequently, the results implied that large tumor size, high histologic grade, and low patient survival rates were associated with high TMEM140 expression levels. Moreover, down-regulation of TMEM140 expression suppressed cell proliferation, migration, and invasion in two glioma cell lines (U87 and U373), which indicated that TMEM140 could be a potential therapeutic target of gliomas and warrants further investigation.

Unstrained cell proliferation is one of the most remarkable features of malignancies. Abnormal regulation in cell cycle and cell apoptosis frequently results in aberrant cell proliferation in cancer [26–29]. Here, the silencing of TMEM140 by siRNA transfection significantly induced G1-phase arrest (Fig. 3e) and cell apoptosis (Fig. 4), which may trigger the inhibitory effects of TMEM140 siRNA on cell proliferation in vitro and in vivo.

Cell adhesion, migration, and invasion are key steps for tumorigenicity in tumor progression [26, 30–32]. Based on GSEA of the TCGA GBM dataset, we found that TMEM140 was associated with cellular adhesion molecules (Fig. 5a), which have been implicated in tumor progression. In addition, our in vitro data demonstrated that the depletion of TMEM140 significantly suppressed cell adhesion, migration, and invasion (Fig. 5b–d). Furthermore, TMEM140 knockdown remarkably decreased the protein levels of ICAM1, VCAM1, and Syndecan1 (Figs. 5e, f), which are considered to be important in glioma cell migration and invasion [33]. These data provided useful information explaining how TMEM140 siRNA inhibited glioma cell migration and invasion, although more in-depth studies are necessary.

## Conclusion

In conclusion, we found for the first time that TMEM140 accumulates in gliomas. The higher level of TMEM140 is strongly correlated with tumor size, histologic grade, and overall survival time in this disease. TMEM140 silencing could suppress the viability, migration, and invasion of glioma cells. Our data demonstrated that TMEM140 is not only a prognostic biomarker but also a therapeutic target in human gliomas.

## Materials and methods

### Tissue collection

A total of 70 patients with glioma were enrolled in this study. The patients underwent surgical removal of their tumors at Xinhua Hospital, Shanghai Jiaotong University School of Medicine, Shanghai, China, from January 2009 to December 2010. All patients had intracranial gliomas and no history of other malignancies. Histological sections of the primary resected surgical specimens were reviewed by authoritative pathologists, according to the 2007 WHO Classification of Tumors of the Central Nervous System [1]. The patients’ clinical characteristics, such as age, gender, and tumor size, were collected for statistical analysis. All patients had complete 5-year follow-ups until death or until the last follow-up. Overall survival time was calculated from the date of the initial surgical operation until death. Additionally, 14 non-neoplastic brain tissue samples were obtained from surgical procedures for epilepsy. The study protocol was approved by the local, independent ethics committee at Xinhua Hospital, Shanghai Jiaotong University School of Medicine. Written informed consent was obtained from all patients.

### Cell culture

The human glioblastoma cell lines (U87, U251, SHG44, U373, and T98G) were purchased from the Cell Bank of the Shanghai Branch of the Chinese Academy of Sciences (Shanghai, China). All culture media were supplemented with 10 % fetal bovine serum (FBS, Hyclone, Logan, UT, USA) and 100 U/mL penicillin/streptomycin (Gibco, Carlsbad, CA, USA). U87, U373, and T98G were cultured in Eagle’s Minimum Essential Medium (MEM, Hyclone), while the U251 and SHG44 cells were cultured in Dulbecco’s Modified Eagle’s medium (DMEM, Hyclone). All cell lines were maintained in a humidified atmosphere, with 5 % CO_2_ at 37 °C.

### Gene silencing

Two glioma cell lines, U87 and U373, were transfected with siRNA oligonucleotides using Lipofectamine 2000 (Invitrogen, Carlsbad, CA, USA). Briefly, siRNA and Lipofectamine 2000 were incubated separately with Opti-MEM for 5 min and mixed together for 20 min at room temperature, and then the mixture was applied to cells plated in 4 mL of medium (final concentration of siRNA, 60 nM). The siRNA sequences were as follows:

- TMEM140-RNAi-1, 5′-GCAUCAUAGUCCUCGUGAUUU-3′;
- TMEM140-RNAi-2, 5′-AGGGUUCAGUUCCAACCAUUU-3′;
- TMEM140-RNAi-3, 5′- ACCCAGAACUUGGAAAGACUU-3′;
- and normal control scrambled siRNA, 5′- UUGUACUACACAAAAGUACUG-3′.

All siRNAs were purchased from GenePharma (Shanghai, China).

### Immunohistochemical analysis

Tissues, 5-μm sections, were dehydrated and subjected to antigen retrieval and endogenous peroxidase blocking. The sections were subsequently incubated with TMEM140-specific antibody (Abcam, Cambridge, MA, USA) overnight at 4 °C followed by incubated with goat anti-rabbit secondary antibody, and the sections were subsequently developed using 3,3-diaminobenzidine (DAB) solution and counterstained with hematoxylin. The staining assessments were performed by two senior neuropathologists who were blinded to the clinical parameters. The specimens were graded into two groups, according to the extent of positivity, as follows: low, <25 % of the tumor cells showed positive stain; and high, >25 % of tumor cells showed positive stain.

#### Real-time reverse transcription PCR (RT-PCR)

Total RNA was extracted from the glioma and normal brain tissues or from the cell lines using TRIzol reagent (Invitrogen), according to the manufacturer’s instructions. DNase I-treated RNA was reverse transcribed using a first-strand cDNA synthesis kit (Fermentas, Hanover, MD, USA). Each cDNA was amplified using a standard SYBR Green kit (Thermo Fisher Scientific, Rockford, IL, USA) and then loaded into the 7300 RT-PCR Detection System (Applied Biosystems, Foster City, CA, USA). The thermal cycling conditions were as follows: 95 °C for 10 min for the first step, and then for ensuing 40 cycles, 95 °C for 15 s and 60 °C for 45 s. The following PCR primers were used:

- TMEM140 (NM_018295.4),
- forward primer: 5′-AAACCATGCAGCTCATTGTC-3′;
- reverse primer: 5′-TCGTTTGTTGCCGTAAGTTC-3′;
- GAPDH (NM_001256799.2),
- forward primer: 5′-AATCCCATCACCATCTTC-3′; and
- reverse primer: 5′-AGGCTGTTGTCATACTTC-3′.

### Western blot analysis

Cell lysates were extracted with RIPA cell lysis buffer, and the protein concentration in the lysates was quantified using an enhanced bicinchoninic acid protein assay kit (Thermo Fisher Scientific). Protein samples (30–50 μg) were loaded for immunoblotting using antibodies against TMEM140, Bcl2, Bax, intercellular adhesion molecule 1 (ICAM1), vascular cell adhesion molecule1 (VCAM1) and Syndecan1 (Abcam, Cambridge, MA, USA), and GAPDH (Cell Signaling Technology, Danvers, MA, USA). Specific proteins were detected with enhanced chemiluminescence (ECL, Millipore, Bredford, USA). Band density was measured (ImageJ software) and normalized to GAPDH.

### Tumor formation assay

Five-week-old male athymic nude mice were purchased from the Shanghai Experimental Animal Center. U87 cells transfected with TMEM140-siRNA or the control siRNA for 24 h were trypsinized, resuspended in PBS, and then subcutaneously injected into the flank of nude mice with 2 × 10^6^ cells per injection (*n* = 5 per group). Tumor size was measured with a Vernier caliper every 4 days and calculated as (length × width^2^)/2. All procedures were performed in accordance with the National Institutes of Health Guide for the Care and Use of Laboratory Animals.

### Cell viability assay

Cell viability was assayed using the Cell Counting Kit-8 (CCK-8, Dojindo Laboratories, Tokyo, Japan) according to the manufacturer’s instructions. Briefly, treated and untreated cells were seeded in 96-well plates at a density of 1000–1500 cells per well and incubated for 1, 2, or 3 days. At the indicated time point, the CCK8 solution was added to each well and incubated for 1 h. The absorbance value (optical density) of each well was measured at 450 nm. For each experimental condition, three wells were used. All experiments were performed in triplicate.

#### Evaluation of cell cycle distribution and cell apoptosis by flow cytometry

Treated and untreated cells were harvested and fixed in 70 % ethanol at −20 °C overnight. Fixed cells were then washed in PBS and stained with 0.1 mg/ml propidium iodide (PI, Sigma, St. Louis, MO, USA) containing 1 mg/ml RNase A for 30 min at 37 °C. Intensities of fluorescence signals were measured on a FACScan flow cytometer (BD Biosciences, San Jose, CA, USA). The percentages of cells in the G0/G1, S, and G2/M phases were determined using FlowJo software (Tree Star). All experiments were performed three times.

The percentage of cells actively undergoing apoptosis was determined by double staining with Annexin V-fluorescein isothiocyanate (FITC) and PI. Transfected cells were harvested, double-labeled with Annexin V-FITC and PI apoptosis detection kits (KeyGEN Biotech, Nanjing, China), and analyzed using a FACScan flow cytometry. At least 20,000 cells were acquired for each sample. The experiments were performed in triplicate.

### Cell adhesion assay

Treated and untreated cells were seeded onto fibronectin-coated 12-well plates at a density of 1 × 10^5^ cells per well and allowed to adhere at 37 °C for 1 h. After the non-adherent cells were washed with a PBS, the attached cells were fixed in 4 % paraformaldehyde and stained with GIEMSA solution. The adherent cells were photographed and counted under an Olympus inverted microscope (Lake Success, NY, USA). The experiments were performed in triplicate.

### Cell migration and invasion assays

In vitro cell migration and invasion assays were performed using 24-well Transwell chambers (8-μm pores, Coring Incorporated, NY, USA). The treated and untreated cells were cultured in the top chamber with serum-free media in triplicate at 5 × 10^4^ cells per well. In the lower chamber, 500-μl media with 10 % FBS was added as a chemoattractant. After 24 h of cultivation, the media from the chamber and the Transwell were removed, and the chamber was gently wiped with a cotton swab. The migrated cells were fixed in 4 % paraformaldehyde, stained with crystal violet solution and counted under a microscope in five fields (×200). The procedure for the cell invasion assay was similar to the cell migration assay, except that the Transwell membranes were precoated with Matrigel (BD Biosciences).

### Gene Set Enrichment Analysis (GSEA)

Glioblastoma multiforme (GBM) cohort downloaded from The Cancer Genome Atlas (TCGA) was analyzed by GSEA. GSEA was performed using GSEA software, Version 2.0.1, which was obtained from the Broad Institute (http://www.broad.mit.edu/gsea; ref. 20), as previously described [34–37]. Gene set permutations were performed 1000 times for each analysis. The nominal *P* value and normalized enrichment score (NES) were used to sort the pathways enriched in each phenotype.

### Statistical analysis

All statistical analyses were performed using the SPSS 16.0 software package. Data are presented as the mean ± S.D. A two-tailed Student’s *t*-test was used for between-group comparisons. The correlation between the IHC staining score and clinicopathologic variables was evaluated using a chi-squared test. Kaplan-Meier survival curves were calculated using death as the end point. The difference in the overall survival curves was examined with a log-rank test. Differences were considered to be statistically significant at *P* < 0.05.

### Ethical approval

All procedures performed in studies involving human participants were in accordance with the ethical standards of the local, independent ethics committee at Xinhua Hospital, Shanghai Jiaotong University School of Medicine and with the 1964 Helsinki declaration and its later amendments or comparable ethical standards. Written informed consent was obtained from all patients.

All procedures performed in studies involving animals were in accordance with the ethical standards of the local, independent ethics committee at Xinhua Hospital, Shanghai Jiaotong University School of Medicine.

### Ethics, consent, and permissions

Informed consent was obtained from all individual participants included in the study.

### Consent to publish

Authors have obtained consent to publish from the participant (or legal parent or guardian for children) to report individual patient data. This is required where an article reports an individual participant’s data in any form (including images, videos, voice recordings, etc.).
