# Erratum: TMEM140 is associated with the prognosis of glioma by promoting cell viability and invasion

**DOI:** 10.1186/s13045-015-0199-0

**Published:** 2015-09-02

**Authors:** Bin Li, Ming-Zhu Huang, Xiao-Qiang Wang, Bang-Bao Tao, Jun Zhong, Xu-Hui Wang, Wen-Chuan Zhang, Shi-Ting Li

**Affiliations:** Department of Neurosurgery, Xinhua Hospital, Shanghai Jiaotong University School of Medicine, Shanghai, 200092 China; Department of Oncology, Fudan University Shanghai Cancer Center, Shanghai, 200032 China

It has been brought to our attention that in Fig. two of the article [1], the immunoblots in the middle panel were incorrectly labeled as TREM2 instead of TMEM140. The correct figure is included below (Fig. 1), in which the immunoblots in the middle panel are labeled as TMEM140. This labeling correction does not affect the results or interpretation of the results. We apologize for this error.

**Fig. 1:**
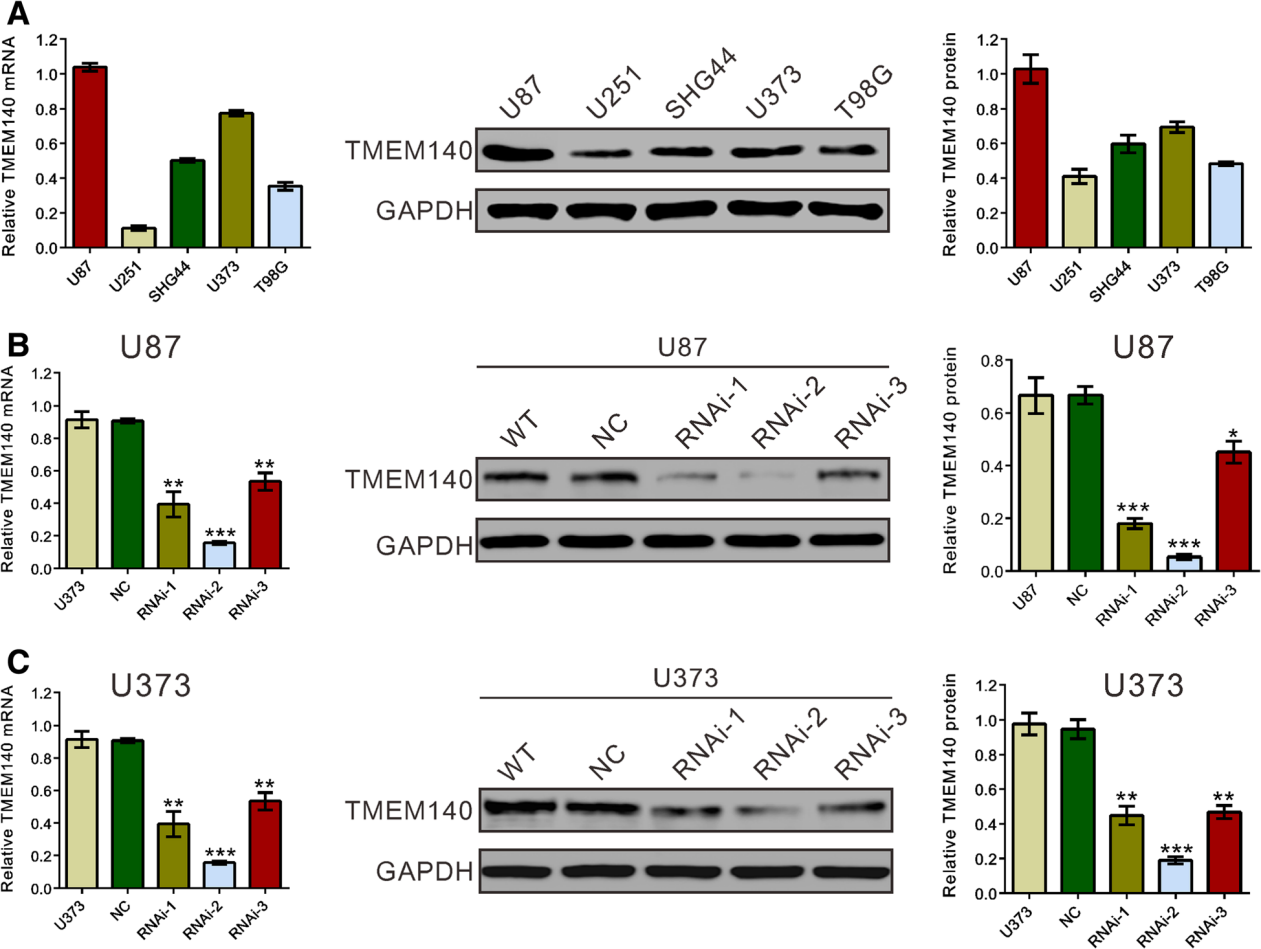
Suppressing of TMEM140 expression by RNAi. **a** TMEM140 expression level in five glioma cell lines was analyzed by RT-PCR (*left*) and immunoblot (*middle* and *right*). **b**, **c** The effect of TMEM140 knockdown through siRNA silencing. The cells were transfected with normal control or TMEM140-RNAi for 48 h and then subjected to RT-PCR (*left*) and immunoblot analysis (*middle* and *right*) of the TMEM140 expression level. The representative images for immunoblot are shown in the *middle panel*, and data from three independent experiments were expressed as the mean ± S.D. (*right panel*). Wild type: wild-type cells; normal control: scrambled siRNA transfected cells; RNAi-1, RNAi-2, and RNAi-3: TMEM140-RNAi-1, -2, and -3 transfected cells (*P < 0.05, **P < 0.01, ***P < 0.001 compared with normal control)
